# Global challenges for lens and cataract research

**DOI:** 10.3389/fopht.2026.1949916

**Published:** 2026-09-14

**Authors:** Michael Wormstone

**Affiliations:** Research Centre for Life Sciences and Healthcare, Nottingham Ningbo China Beacons of Excellence Research and Innovation Institute, University of Nottingham, Ningbo, China

**Keywords:** cataract, cataract surgery, experimental models, lens, posterior capsule opacification, presbyopia

## Abstract

The lens is sadly too often dismissed as a less important tissue within the eye, but this neglects to appreciate the role it plays in the wellbeing and quality of life of individuals, the economic burden lens pathologies place on society and the true biological wonder it is! A normal healthy lens in isolation, looks like a jewel, formed from biological glass. The lens is made of cells enveloped in a soft collagen shell. No blood supply, no nerves. There is slow cell growth that occurs progressively over a lifetime and little cell death takes place. Furthermore, the lens is not a static system and has to quite literally accommodate and manage ongoing mechanical changes due to shape-changing accommodation. Despite these challenges, lens cells are orchestrated to perfect harmony to generate an anatomy conducive to transparency, refraction and accommodation. Pathologies of the lens compromise wellbeing. Congenital and early onset cataract can lead to blindness in tens of thousands of people, while in later life age-related cataracts lead to visual deterioration and blindness in tens of millions. The only treatment for cataract is surgery with intraocular lens (IOL) implantation. Cataract surgery is the most common operation in the world and IOLs the most common medical implant. Despite the success of cataract surgery, wound-healing responses lead to secondary visual loss, known as posterior capsule opacification, in approximately 20% of patients. This can be resolved by Nd: YAG Capsulotomy, but this is not without risk; this is the second most common operation in the world. In addition, presbyopia commonly present in middle age and affects billions, impacting personal wellbeing and ability to perform specific roles. Collectively, these conditions compromise individuals and place significant financial burdens on healthcare providers. The need to undertake lens and cataract research therefore remains a key health priority. As a community the lens field faces both medical and political challenges that need to be considered, appreciated and addressed. In the current article the lens and cataract research landscape is assessed and evaluated with potential strategies proposed to build on existing strengths and address areas of underused potential to continue advancing knowledge and impact on the health and wellbeing of millions and potentially billions of patients.

## 1 Introduction

The lens is a pivotal point in our visual system. It is often perceived to be a simple tissue, which at first glance may be apt, but the organisation of cells and structures within it need to be coordinated with exquisite control and order to maintain transparency and refract light to a focus on the retina (1, 2). This is a remarkable achievement for a living tissue! The lens should therefore be considered a biological marvel that should be celebrated and better understood to reveal fundamental biological questions and improve the wellbeing of millions of patients who develop lens related pathologies (3–9) (**Figure 1**). In the following text, I will highlight the incidence and significance of lens pathologies (**Figure 1**) and present a coordinated approach to future research from basic discovery to translation (**Figure 2**).

**Figure 1:**
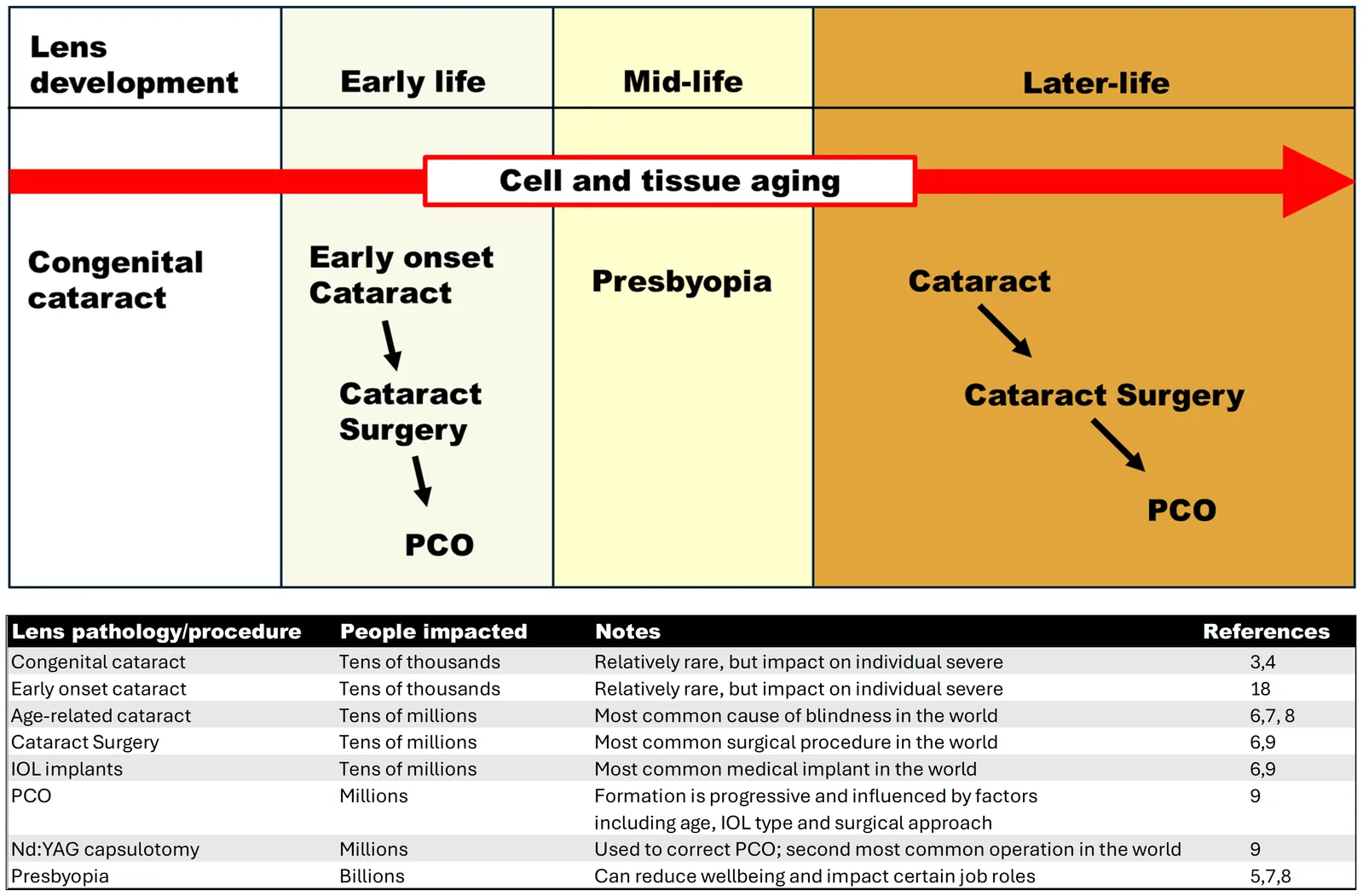
Association of lifespan and lens-associated pathologies (Top). A list of key lens-associated pathologies/procedures and their scale of impact on people (Bottom).

**Figure 2:**
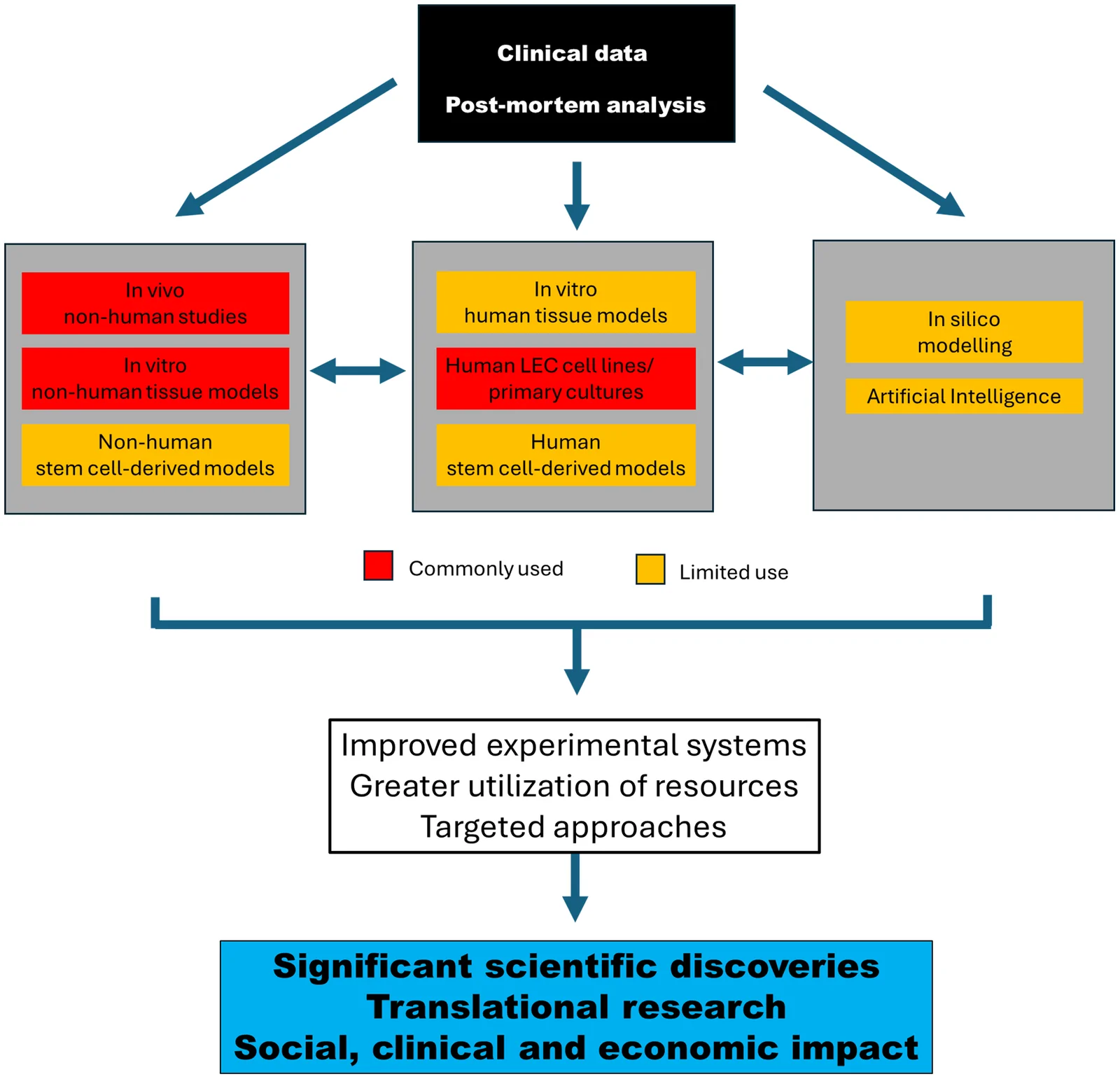
A schematic integrated workflow for lens and cataract research.

## 2 Lens pathologies across the lifespan

To highlight significant interests in the lens it is worth following a timeline from the embryo through to later life.

The developmental stages of the eye and lens within the embryo are well characterised and exhibit some degree of conservation across species in addition to divergent paths (10, 11). Multiple model systems have been developed that permit controlled gene regulation, which enables key regulatory systems to be determined across specific time signatures in development (12). Such work has informed the critical importance of the lens in ocular tissue organisation and key factors driving lens generation. Continued understanding of these fundamental processes can provide insight to congenital disorders and support research activity on the mature lens.

From the point at which the lens vesicle forms in embryogenesis, the lens in most cases, becomes relatively isolated, such that lens cells remain contained within their collagen envelope (12). This separation is even more remarkable when one appreciates that the lens is not typically served by blood vessels or nerves and is bathed by the aqueous and vitreous humours. Under normal circumstances this setting would be detrimental to cell wellbeing and compromise tissue maintenance. However, the lens is a resilient tissue, and its cells have adapted to cope with this challenging environment. Due to the presence of the capsule, the lens is fundamentally a bound tissue (13). Consequently, there is little to no turnover of cells within the lens. Cell division, limited to the equatorial region, is slow and steady and death negligible (14). Quite simply, there is nowhere for excess material to go, whereas phagocytosis can address cell death to a limited extent. The lens continues to differentiate from epithelial cells to fibre cells, which results in increased mass and to a lesser degree volume (15). The newest fibre cells reside at the periphery while the central core is derived from embryonic fibre cells. The bulk of the epithelium has also been present since birth and thus the cells are as old as we are. It is remarkable that the lens maintains clarity and refractive powers for so long and achieves this through a combination of exquisite cell organisation and adaption. The lens functions as a syncytium (1, 2), such that the cells are connected to behave as a giant cell, while many of the survival factors detected in the surrounding environment are produced by the lens (16, 17). The lens is therefore a wonderful study model for aging of both individual cells and a tissue.

While cataract can appear prematurely due to injury (18) or disease association e.g. Werner’s syndrome (19), myotonic dystrophy (20), its incidence is relatively low and, in most cases, the lens functions well in the early stages of life. Conditions such as myopia and hyperopia are a consequence of imbalance between the refractive system within the eye and its size and can be overcome by refractive adaptation e.g. glasses, contact lenses or refractive surgery. However, in middle-age consequences of biological events within the lens give rise to presbyopia, which affects billions of people (5, 7, 8). Presbyopia impacts on our ability to see near objects. This develops due to the lens becoming less flexible and unable to change shape adequately during accommodation. Consequently, the lens is unable to meet the refractive needs to see objects close by, clearly. This is a consequence of the progressive growth and aging of the lens and if uncorrected can impact on daily functions, which can limit performance in some vocations and can have a general impact on quality of life (5). Advances have been made to manage or resolve presbyopia using drug therapy, surgical approaches and refractive correction (5). Nevertheless, the problem remains significant and requires ongoing investigation at the clinic and basic research levels.

Cataract (a clouding of the lens) is the primary pathology of the lens. In 2020, the estimated number of cases classified as blind through cataract in people over 50 years of age, globally was 15.2 million, and a further 78.8 million cases exhibited moderate or severe vision impairment (7, 8). Cataract is a general term, and multiple forms exist that arise through differing mechanisms and demonstrate regional distribution patterns within the lens. The most common and well-studied forms of cataract are nuclear, cortical, anterior subcapsular and posterior sub-capsular cataracts (6). Cataract has a marked impact on the wellbeing and productivity of individuals and carries a major economic cost to healthcare providers (21). Furthering our understanding of lens homeostasis and prevention/delay of cataract formation remain key objectives for lens researchers.

If cataract develops, the only means of treatment is surgical removal of the cataract: this is the most common operative procedure in the world (9). Cataract surgery involves making a circular opening (capsulorhexis) in the anterior lens capsule and removal of lens fibre cells. The product of cataract surgery is known as a capsular bag, which comprises a ring of the anterior capsule and entire posterior capsule. The capsular bag remains *in situ*, partitions the aqueous and vitreous humours, and in the majority of cases, houses an intraocular lens (IOL). Initial results are superb, but a significant number of patients subsequently develop a condition known as posterior capsule opacification (PCO; 9). This arises from wound-healing responses that lead to fibrosis and partial lens regeneration, which collectively cause light scatter within the visual axis. PCO causes significant visual loss in approximately 20% of patients: the only treatment for this is laser surgery to ablate light scattering regions (9). This is a major cost to healthcare providers and carries several risks to the patient, including the risk of retinal detachment. Exploration of the underlying mechanisms driving PCO is key. There are two generally recognised forms of PCO, known as fibrotic and regenerative (9). More research focus has been placed on fibrotic PCO, however there are aspects to this topic that need further consideration and direction. Key features associated with fibrotic PCO include cell growth/migration, matrix deposition, matrix contraction and epithelial-mesenchymal transition (EMT). Undoubtedly Transforming growth factor β (TGFβ) plays a key role in fibrotic PCO and not surprisingly this molecule features in many publications (22–25). However, the notion that mediation of TGFβ actions through Smad-signalling leads to EMT is the cause of PCO is too simplistic and built on assumptions that do not consider the wider knowledge and regulatory complexity of PCO (9). EMT is a process, that does occur in PCO and is strongly regulated by TGFβ, but this in itself does not cause light scattering and visual deterioration. Cell aggregation, matrix deposition and particularly matrix contraction are responsible for reduced visual quality in fibrotic PCO (26). The conventional assumption is that EMT is a pre-requisite for contraction, however some reports suggest TGFβ-induced matrix contraction does not require the EMT marker αSMA, Fibronectin or Smad4 dependent signalling (27, 28). The general understanding of regulatory systems is more complex than commonly assumed and requires further understanding of growth factors driving cell growth/migration and greater understanding of TGFβ/Smad-independent signalling in the wider regulation of PCO characteristics. I therefore feel that too much weight is currently placed on EMT as a readout and, when possible, a wider range of fibrotic PCO characteristics, particularly matrix contraction/wrinkling should also be investigated to establish a clearer picture of regulatory mechanisms and improve screening of potential therapeutic candidates. While the majority of research effort in the field has been directed to fibrotic PCO, regenerative PCO is important. Features of regenerative PCO, such as Soemmerring’s ring and in particular Elschnig’s pearls can lead to light scatter and notable visual deterioration (9, 26). Greater analysis of Soemmerring’s ring and Elschnig’s pearls is needed to establish better experimental models of regenerative PCO, which is an area requiring development and research effort (9). Parallel development of intraocular devices through design and material advances in combination with selected therapeutic treatments are likely to give the best opportunity for long-term prevention of PCO.

## 3 Advancing lens and cataract research

The lens research community is made up of talented people with a range of complimentary skill sets. It is, however, a pool of talent that needs to grow and welcome, entice and retain new members to add their flavour to the mix. As funding restrictions limit opportunities to study the lens, the field and its members are in danger of becoming isolated and disconnected. The question, therefore, is how we can redress this trend and establish a platform to stabilise and grow.

The days of academic freedom are to some degree an ideal of science past. Funding is too tight and while we should not stifle creativity and innovation this has to be channelled towards a specific purpose with tangible goals and impact. Some of the work will be an important scientific advance, while in other cases outcomes may enable translational research with benefits to industry, clinicians or patients. In terms of messaging, it is important to define what our work will achieve and to the benefit of whom (industry, scientists, patients). Regarding medical impact, in the vast majority of cases our target beneficiaries are human patients (though we should not forget animals can also be patients too) (29). If research work is orientated to health benefits it is important to demonstrate how work on an animal (*in vivo* or *in vitro*), cell line or any other model relate to or can predict outcomes in patients. No model system other than a human being truly predicts outcome, so we need to be realistic about the strengths and limitations of experimental platforms available.

When I review the lens and cataract research landscape, the majority of publications utilise *in vivo* or *in vitro* animal models and lens cell lines (generally of human origin) (30–32). This is understandable based on accessibility and existing expertise. Such approaches will continue to play an important role in research programmes, but the question that needs to be asked is, are these resources being used optimally to advance human health? The more human data we obtain, the greater cross-referencing with experimental models can take place and thus determine relevance of a given model for a specific research question. This also includes making better use of historic data. In the days before modern cataract surgery whole human lenses were removed at surgery (33) and were analysed (34–36); this analysis was often biochemical, physical or physiological rather than modern molecular tools. It is therefore important not to ignore past findings, but to harness their power and marry the data with new discoveries. The principle of greater utilisation of human data also extends beyond basic research and is relevant to drug development and cytotoxicity testing (37, 38). There are common features and differences in lenses across species that need greater characterisation to help predict side-effects or actions in human patients. This can inform appropriate selection of pre-clinical testing. Such strategies will address the 3Rs (Replacement, Refinement and Reduction) and challenge existing ethical considerations to improve efficiency of drug development.

It has always surprised me that as a community we do not utilise an incredibly precious resource, namely human donor cadaver eyes, as much as we should. While it is still employed successfully (15, 34, 38–45), I strongly believe that greater utilisation of this precious material, so kindly donated for research, will strengthen the collective research effort and lead to accelerated advances in scientific discovery and clinical outcomes. Of all tissues in the body, the lens is arguably the most suitable for post-mortem culture. This material can be characterised using a range of techniques to assess physical, biochemical and molecular profiles (15, 39, 40) to provide baseline reference information for the lens community. Moreover, due to the wide age-spectrum of donors, such patterns may also be observed across different stages of life (15, 27, 41, 42). Post-mortem time still matters and the shorter duration prior to analysis the better. I have personal experience working with human cadaver eyes over three decades in research and have noted that the lens typically has a slower rate of degeneration post-mortem than other cell and tissue types within the eye. Generally, I would estimate the window of opportunity for whole lens culture (43) to be sub-24 hours, while tissue culture models (capsular bag, explants) (44–46) are still feasible up to 96 hours post-mortem; success is also influenced by multiple factors, such as enucleation time, dissection quality, appropriate storage and transport.

The body of work available that has utilised human tissue, while limited in volume, has generated significant findings that can guide research paths. The issue here is why there isn’t greater uptake and utilisation of human donor eyes? Is it limited by access to material? Time to receive material? Is the quality of the material provided good enough? Associated costs? Difficulty with ethical approval processes? Potentially a researcher interested in developing a programme involving human cadaver eyes may meet one or several of these issues. If the obstacles appear too great understandably many will grow frustrated and not pursue this option further. In addition, legislation and practices may differ across regions/countries. Nevertheless, integration of human tissue to a laboratory’s research programme adds great value and enables self-validation of findings against other experimental systems used in the laboratory. The ideal scenario is to establish a local working partnership between lab and hospital/Eye bank to provide material for research projects with full ethical approval. If this can be established, post-mortem times are reduced, transport is limited and generally the tissue preservation should be optimal and increase the scope of experimental procedures. The next best option is a provincial/state level source for large countries or national bases for smaller nations; however, this will extend post-mortem times. While in the UK, I experienced both scenarios and each are feasible options. Maintaining a clear dialogue and line of communication is vital to ensure both parties understand what they need to do to ensure the generous donation is best prepared and utilised for research endeavours. Eye banks are ubiquitous across the world and thus the potential to draw on this resource, in principle, is available to most lens and cataract researchers. In the USA, a recent paper suggested that restrictions on donor tissue for research is limited by agreed post-mortem times and staff working hours (47). Depending on the planned experiments, a lens researcher could request longer post-mortem time limits, while shift patterns could be adopted by eye bank teams to increase retrieval windows. In fact, it has been suggested that extending the general working day at an eye bank by 3 hours to incorporate peak donation times and increasing post-mortem intervals from <12 hours to <16 hours would increase research tissue availability by 223% (47).

While individual pursuits are worthwhile, a unified message is stronger. I would therefore advocate developing co-ordinated efforts to seek central government support to improve accessibility to eye banks and clinical samples, reduce costs and ensure tissue quality. This would involve local networks/partnerships between eye banks and research centres/labs. If such co-operatives can be established across nations, international programmes can be established, and global impact can be borne from concerted and co-ordinated effort.

I also wish to highlight further research streams that I feel are under-utilised. Stem cells have become an incredibly valuable tool for research relating to cell types and tissues throughout the body. The work by Yang et al. (48) opened-up this experimental stream to the community and while others have built on this platform to make significant contributions (9, 49–51) the field engagement by the wider lens and cataract research pool has been limited. Moving forwards, greater characterisation of lens organoids relative to human tissue will serve as a marker of progress and can guide culture protocols and increase utility of stem cell-derived lens organoids for lens research applications and drug screening (49, 51).

Computational modelling and artificial intelligence have evolved dramatically in recent years as processing systems and hardware have advanced (52–56). The introduction of in silico approaches can help answer a host of questions in the lens research field to help understand accommodative changes, refractive patterns, IOL-capsular bag interaction and more (52, 53). AI is being integrated into the lens land cataract field in many ways, which include cataract grading, surgical training, IOL power calculations, and biological discovery (54–56). AI requires a training phase followed by verification, refinement and general application. Consequently, the foundation of both AI and computer modelling is robust data. In the majority of cases, model designs are human oriented. While animal data can serve to develop the system, it may not predict human behaviour. Available human data is being used to build and validate these systems, but further human-derived data is required to better support and guide future developments.

## 4 Summary

The lens is a truly important tissue that presents an incredible study platform for basic science. Pathologies of the lens impact on millions, even billions of people worldwide, which reduce individual quality of life and impose financial burden to healthcare providers. The lens research community is comprised of talented people, but we need to collectively build on our existing strength and develop underused resources in a concerted and coordinated manner to provide a stable platform for lens and cataract research to grow and improve the lives of many.

## Data Availability

The original contributions presented in the study are included in the article. Further inquiries can be directed to the corresponding author.
